# Unveiling the Genetic Complexity of Teratozoospermia: Integrated Genomic Analysis Reveals Novel Insights into lncRNAs’ Role in Male Infertility

**DOI:** 10.3390/ijms241915002

**Published:** 2023-10-09

**Authors:** Maria-Anna Kyrgiafini, Themistoklis Giannoulis, Alexia Chatziparasidou, Nikolaos Christoforidis, Zissis Mamuris

**Affiliations:** 1 Laboratory of Genetics, Comparative and Evolutionary Biology, Department of Biochemistry and Biotechnology, University of Thessaly, Viopolis, Mezourlo, 41500 Larissa, Greece; 2 Laboratory of Biology, Genetics and Bioinformatics, Department of Animal Sciences, University of Thessaly, Gaiopolis, 41336 Larissa, Greece; 3 Embryolab IVF Unit, St. 173-175 Ethnikis Antistaseos, Kalamaria, 55134 Thessaloniki, Greece

**Keywords:** male infertility, teratozoospermia, long-noncoding RNA (lncRNA), variant

## Abstract

Male infertility is a global health issue, affecting over 20 million men worldwide. Genetic factors are crucial in various male infertility forms, including teratozoospermia. Nonetheless, the genetic causes of male infertility remain largely unexplored. In this study, we employed whole-genome sequencing and RNA expression analysis to detect differentially expressed (DE) long-noncoding RNAs (lncRNAs) in teratozoospermia, along with mutations that are exclusive to teratozoospermic individuals within these DE lncRNAs regions. Bioinformatic tools were used to assess variants’ impact on lncRNA structure, function, and lncRNA–miRNA interactions. Our analysis identified 1166 unique mutations in teratozoospermic men within DE lncRNAs, distinguishing them from normozoospermic men. Among these, 64 variants in 23 lncRNAs showed potential regulatory roles, 7 variants affected 4 lncRNA structures, while 37 variants in 17 lncRNAs caused miRNA target loss or gain. Pathway Enrichment and Gene Ontology analyses of the genes targeted by the affected miRNAs revealed dysregulated pathways in teratozoospermia and a link between male infertility and cancer. This study lists novel variants and lncRNAs associated for the first time with teratozoospermia. These findings pave the way for future studies aiming to enhance diagnosis and therapy in the field of male infertility.

## 1. Introduction

Infertility is a significant global health concern that affects approximately 50 million couples worldwide [1]. It is estimated that around 12.5% of women and 10% of men experience infertility [2]. The World Health Organization (WHO) defines infertility as the inability to achieve pregnancy after engaging in regular unprotected sexual intercourse for a duration of 12 months or longer. The male factor contributes to 50% of infertility cases [3], and various sperm defects, such as quantity and quality issues, can be identified through semen analysis as causes of male infertility [4], though the etiology of sperm defects remains idiopathic in 30–50% of cases [5]. Teratozoospermia is defined as a percentage of morphologically normal spermatozoa below the lower reference limit. However, the definition of normality has significantly changed over the past few decades, ranging from 50% in the initial WHO classification in 1980 [6] to 4% in the most recent version published in 2010 [7]. In general, a morphologically normal spermatozoon is characterized by specific features, including a normal acrosome, an oval-shaped head with a width ranging from 2.5 to 3.5 μm and a length between 5 and 6 μm, a midpiece measuring approximately 4.0 to 5.0 μm, and a tail that is approximately 50 μm in length [8]. Teratozoospermia encompasses a heterogeneous group of abnormal sperm phenotypes that can affect different parts of sperm, such as the head, neck, midpiece, and tail. These abnormalities may occur individually or concurrently [9].

Although significant progress has been made in investigating teratozoospermia, the molecular mechanisms underlying this condition in male infertility are not yet fully understood. In general, the identification of the molecular causes of male infertility is a major challenge. It is estimated that more than 4000 genes play a role in spermatogenesis, making the characterization and identification of their specific contributions a complex undertaking [10]. Furthermore, emerging evidence suggests that noncoding RNAs have crucial Molecular Functions [11], including a role in reproduction and male infertility [12]. Various techniques, such as real-time quantitative PCR, microarrays, and high-throughput next-generation sequencing, have been developed to detect and measure noncoding RNAs. These techniques provide valuable insights into the role of noncoding RNAs and the development of complex diseases, such as cancer, diabetes, cardiovascular diseases, etc. [13,14]. Regarding reproduction, attention has been drawn to a particular type of noncoding RNA, long-noncoding RNAs (lncRNAs). LncRNAs, a diverse group of RNA molecules larger than 200 nucleotides, despite lacking the ability to code for proteins, play important roles in cellular functions. They can be classified based on their chromosomal location in several categories, including antisense lncRNAs, intronic lncRNAs, divergent lncRNAs, intergenic lncRNAs, promoter-associated lncRNAs, transcription start site-associated lncRNAs, and enhancer RNAs [15]. LncRNAs also exhibit four distinct functions [15,16,17,18]: signaling, where they regulate gene transcription either independently or with proteins like transcription factors; decoying, in which they bind to proteins to impede interactions with DNA, mRNA, or miRNA; guiding, as they transport proteins to precise intracellular sites; and scaffolding, where they facilitate the assembly of macromolecular complexes for coordinated interactions.

When it comes to the involvement of lncRNAs in male infertility, particularly teratozoospermia, studies prove that these molecules play a role in the functions of spermatogonial stem cells, including the regulation of their differentiation, proliferation, and self-renewal processes [19]. Moreover, various studies have highlighted distinct expression patterns of lncRNAs between fertile and infertile men [12]. Nevertheless, our understanding of how lncRNAs precisely regulate reproduction and their association with teratozoospermia is still limited and subject to ongoing debate [20]. Except for studies reporting lncRNAs that are deregulated in teratozoospermia, there is also a significant knowledge gap in studying variants and mutations within lncRNA regions and investigating their role in teratozoospermia. Traditionally, research has primarily focused on genetic variations occurring within protein-coding regions to elucidate the molecular mechanisms underlying disease [21]. However, some genome-wide association studies (GWAS) have provided evidence suggesting that polymorphisms within lncRNA genes are linked to human diseases [21,22]. More specifically, single nucleotide polymorphisms (SNPs) in lncRNAs can have various consequences [21]. Firstly, variations in regulatory regions, such as transcription binding sites or lncRNA gene promoters, can affect lncRNA expression, resulting in the dysregulation of associated pathways [22,23]. SNPs can also disrupt the secondary structure of lncRNAs, thereby impacting their interactions with miRNAs, RNA-binding proteins, and other molecules [21,24]. Furthermore, the presence of a variant within the sequence of a lncRNA can potentially affect RNA turnover by altering the binding of proteins responsible for lncRNA’s stability [21]. Lastly, similar to protein-coding genes, mutations in lncRNA regions can disrupt the splicing process and influence the structure and functionality of lncRNAs [21]. Numerous studies have demonstrated that variations in lncRNAs can contribute to the development of various diseases, such as different types of cancer [21,25] and a type of muscular dystrophy [26], through the mechanisms described above. However, despite indications of the significant role of lncRNAs in male infertility, no studies have been conducted to explore whether specific variants can impact lncRNA’s expression or function, thereby leading to specific subtypes of male infertility, such as teratozoospermia.

Thus, this study aims to explore the role of lncRNA variants in the pathogenesis of male infertility, specifically teratozoospermia. To achieve this, we utilized whole-genome sequencing (WGS) data and integrated these with RNA expression profiles from normozoospermic and teratozoospermic men. Our objectives are to (a) identify and characterize variants that present solely in teratozoospermic men and map to differentially expressed lncRNA regions and (b) investigate the impact of prioritized variants on the function, structure, and interactions of lncRNAs, particularly with microRNAs (miRNAs). By exploring these effects, we aim to shed light on the role of lncRNAs in teratozoospermia. The ultimate goal of this study is to provide a valuable reference for future research on teratozoospermia, as identified variants could hold significant importance in unraveling the genetic basis of teratozoospermia, potentially leading to improved diagnosis and treatment.

## 2. Results

### 2.1. WGS and RNA Expression Results—Identification of Exclusive Variants on DE lncRNAs

The primary objective of this study was to identify exclusive variants in teratozoospermic men that are mapped to differentially expressed (DE) lncRNA regions and explore their role in the pathogenesis of teratozoospermia as well as their impact on lncRNAs. To accomplish this, whole-genome sequencing (WGS) and RNA expression data were integrated.

Specifically, 617,722 variants were found exclusively in teratozoospermic men, while 2,342,243 variants were present only in normozoospermic men. The identified variants were mapped to 34,603 and 22,022 genes and characterized as non-coding regions, such as miRNAs and lncRNA genes, in normozoospermic and teratozoospermic men, respectively. However, for this study, only the variants identified exclusively in teratozoospermic men were selected for further analysis, as the aim was to detect and investigate variants in lncRNA regions contributing to teratozoospermia.

Furthermore, to identify lncRNAs that are deregulated in teratozoospermia and, thus, that may play a role in male infertility, the analysis of Zhou and Wang (2020) [27] was utilized. Zhou and Wang (2020) [27], by applying a significance threshold of *p*-value < 0.05 and a fold change cutoff of [logFC] > 2, detected a total of 101 differentially expressed lncRNAs. Specifically, in comparison with the normozoospermic group, 68 lncRNAs were upregulated, and 33 lncRNAs were downregulated in the teratozoospermic group. Supplementary Table S1 provides a comprehensive list of these differentially expressed lncRNAs, as reported by Zhou and Wang [27].

Finally, the two datasets mentioned above, namely the differentially expressed (DE) lncRNAs and the exclusive variants in teratozoospermic individuals, were integrated. The aim was to identify exclusive variants found in teratozoospermic men that were mapped to DE lncRNAs. These variants have the potential to impact the structure, function, and interactions of lncRNAs, leading to altered gene expression and potentially contributing to the pathogenicity of teratozoospermia, as described in the Introduction [21]. Moreover, they have the potential to be used as biomarkers in teratozoospermia. Consequently, a total of 1166 variants found only in teratozoospermic men were identified to be mapped onto DE lncRNAs. The complete list of these unique variants mapped onto DE lncRNAs can be found in Supplementary Table S2. Subsequently, these variants were prioritized by applying a series of filters to investigate their consequences on lncRNAs and their role in teratozoospermia.

### 2.2. Exclusive Variants with a Functional Role

First, to comprehensively evaluate the impact of identified variants on lncRNA functionality and prioritize them, our analysis incorporated data from both RegulomeDB [28] and 3DSNP [29] databases. RegulomeDB [28] provides valuable insights into the regulatory significance of these variants by assessing their association with known regulatory elements, such as transcription factor binding sites and DNase I hypersensitive sites. On the other hand, the 3DSNP [29] database assesses variant significance by considering multiple factors, including thermodynamic stability, evolutionary conservation, protein binding sites, and structural dynamics. Therefore, in our study, we set stringent criteria, considering variants with a 3DSNP score greater than 10 and a RegulomeDB Rank ranging from 1a to 2c. Applying these criteria, we identified 64 variants on 23 lncRNAs that displayed a strong likelihood of influencing lncRNA functionality and contributing to teratozoospermia. These findings are presented in Table 1.

### 2.3. Exclusive Variants Affecting lncRNAs’ Structure

Among the 1166 variants exclusively observed in teratozoospermic men and identified within DE lncRNAs, the variants affecting the secondary structure of lncRNAs were prioritized, as they could contribute to the development of teratozoospermia. Even minor changes in the nucleotide sequence, such as single nucleotide polymorphisms (SNPs), have the potential to disrupt the thermodynamic stability of RNA secondary structures. Consequently, these alterations can impact the overall three-dimensional conformation and stability of lncRNAs, potentially influencing their interactions with other molecules, including proteins and DNA. Furthermore, since lncRNAs lack protein-coding capacities and rely on their structural integrity for proper functioning, SNPs can induce significant structural alterations that can affect the functionality of lncRNAs [30,31,32]; thus, they were prioritized.

In this study, variants with a *p*-value < 0.2, a cutoff indicating an impact on lncRNAs’ structure as determined by lncRNASNP v3 [33], were selected. Overall, the analysis revealed 7 SNPs that affected the structure of 4 specific lncRNAs, as summarized in Table 2.

### 2.4. Exclusive Variants Affecting miRNA–lncRNA Interactions and Investigation of Target Genes of Affected miRNAs

Exclusive variants on teratozoospermic men were also prioritized to identify those that affect miRNA–lncRNA interactions. These variants can lead to the gain or loss of miRNA target sites within lncRNAs, consequently altering the regulation of gene expression [34]. Through their intricate interactions, miRNAs and lncRNAs orchestrate important cellular processes and can contribute to the development and progression of various diseases [35], such as teratozoospermia. Therefore, according to the lncRNASNP v3 database [33], out of the 1166 exclusive variants identified on DE lncRNAs, 37 SNPs were found to affect interactions between 17 lncRNAs and 151 miRNAs. The complete list of variants and the corresponding affected interactions (lncRNAs and miRNAs) can be found in Supplementary Table S3.

Subsequently, an investigation was carried out to determine the overlap of target genes among affected miRNAs, aiming to identify common pathways that become deregulated in teratozoospermia due to lncRNA variants. According to miRTargetLink 2.0 [36], affected miRNAs targeted a total of 198 common genes. The complete list of these genes and their interactions with miRNAs is provided in Supplementary Table S4. It is important to note that only strong and validated interactions were selected for constructing miRNA–mRNA interaction networks.

**Figure 1:**
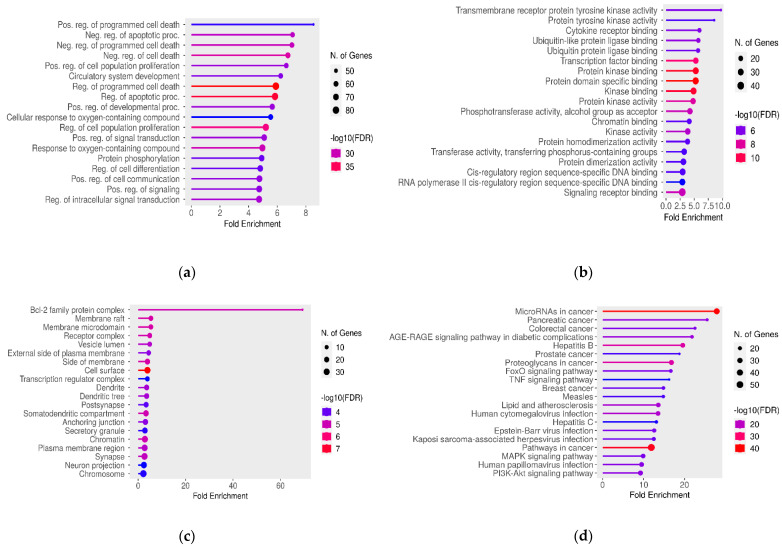
Significant (**a**) GO Biological Process, (**b**) GO Molecular Function, (**c**) GO Cellular Component, and (**d**) KEGG pathway terms associated with the overlap gene targets of miRNAs that are affected by variants in lncRNA regions. The size and color of the dots represent the number of genes and the range of statistical significance, respectively. The red color indicates higher -log10(FDR) values, followed by pink, purple and blue colors. The *y*-axis represents the GO and KEGG terms, and the *x*-axis represents the fold enrichment. The *p*-values were corrected for multiple tests using the false discovery rate (FDR).

Additionally, to gain further insights into the role of target genes, Gene Ontology (GO) Enrichment analysis [37,38] and KEGG pathways analysis [39] were performed. These analyses revealed dysregulation in processes such as cell proliferation and programmed cell death, with many miRNAs and miRNA target genes being involved in pathways associated with various types of cancer. In terms of Cellular Components, most of these genes were associated with the Bcl-2 family protein complex, while the most enriched category for the Molecular Function consisted of genes encoding proteins with tyrosine kinase activity (Figure 1a–d).

### 2.5. Variants Affecting Both Structure or Function of lncRNAs and miRNA–lncRNA Interactions

Then, we identified prioritized variants that had potential effects on both the function or structure of lncRNAs and on miRNA–lncRNA interactions. These variants, which are listed in Table 3, are particularly significant as they are exclusively found in teratozoospermic men and are mapped to lncRNAs, which are known to be deregulated in teratozoospermia.

### 2.6. lncRNAs with Multiple Variants Affecting Their Function, Structure, or Interactions with miRNAs

As a final step in this study, we identified lncRNAs with multiple prioritized variants that affect both the function or structure of lncRNAs and their interactions with miRNAs. The lncRNAs are presented in Table 4. These findings shed light on the potential mechanisms underlying teratozoospermia and provide valuable insights into the role of lncRNAs in male fertility issues.

## 3. Discussion

Previous studies have demonstrated that variants in lncRNAs can impact their function, structure, and interactions with other molecules [21]. Consequently, these variants may lead to the dysregulation of gene expression and contribute to disease development [14,21,40]. While the functions of the majority of estimated ~20,000 lncRNAs remain unknown [21], studies suggest that they play an essential role in spermatogenesis regulation [19,20]. Furthermore, lncRNAs have been associated with male infertility and its specific subtypes [12], such as teratozoospermia, which is characterized by morphological abnormalities in spermatozoa. However, no studies have investigated the consequences of lncRNA variants and their role in teratozoospermia. Therefore, this study aimed to combine WGS and RNA expression profile data to identify exclusive variants found only in teratozoospermic men that are also mapped to lncRNAs with a deregulated expression in teratozoospermia. Subsequently, this study assessed the impact of these variants on the structure, functionality, and interactions of lncRNAs. In summary, out of the 1166 exclusive variants identified, 64 variants on 23 lncRNAs displayed a strong likelihood of influencing lncRNA functionality. Additionally, 7 SNPs affected the structure of 4 lncRNAs, while 37 SNPs were found to affect the interactions between 17 lncRNAs and 151 miRNAs. The affected miRNAs’ overlapping target genes were primarily associated with cancer and processes such as cell proliferation, apoptosis, programmed cell death, etc.

### 3.1. Impact of Variants on the Functionality of lncRNAs

At first, we assessed the impact of variants on the functionality of lncRNAs. Among the variants identified with potential functional roles, only one had previously been associated with a specific disease. Specifically, the variant rs3217986, mapped on *CDKN2B-AS1 (ANRIL),* has been associated with cutaneous melanoma [41] and coronary artery disease [42]. Regarding the other lncRNAs on which these variants were mapped, *LINC00592* has been demonstrated in several studies to play a role in various cancer types through transcription regulation [43,44]. Other lncRNAs identified as potentially involved in cancer include *FRMD6-AS1* [45,46] and *LINC00466* [47,48], which have also been shown to promote tumor growth in vivo alongside [49] *LINC00877* [50], *LINC01091* [51], *NNT-AS1* [52,53,54], *ZNF252P-AS1* [55], *LZTS1-AS1* [56], and *OTUD6B-AS1* [57,58]. Of particular significance is also the fact that the host gene of *NNT-AS1*, *NNT*, encodes an inner mitochondrial membrane protein and produces large amounts of NADPH. *NNT* deficiency has been reported to cause complete germ line loss and azoospermia [59]. Additionally, a separate study has associated *NNT* mutations with impairments in gonadotropic function and genitalia [60].

### 3.2. Impact of Variants on the lncRNAs’ Structure

Variants can also have significant effects on the structure of lncRNAs. These alterations can disrupt stable secondary structures within lncRNAs, potentially impacting base-pairing interactions and binding sites for other molecules, or can influence alternative splicing, resulting in distinct structural conformations of lncRNA isoforms [30,31,32,61]. It should be noted that any of the structural alterations described above can significantly impact functionality. These changes can alter binding affinity, subcellular localization, stability, and the accessibility of functional domains within lncRNAs. Consequently, they can influence lncRNAs’ role in gene regulation and cellular processes. In the present study, none of the variants identified to affect lncRNAs’ structure had been previously associated with male infertility or other diseases. Regarding the lncRNAs on which these were found, *PRMT5-AS1* is highly expressed in the testis, but its role and function have not been extensively studied. Baytak et al. (2017) [62] suggested its involvement in lymphoma and its potential impact on cell growth. Interestingly, however, its host gene, *PRMT5*, is essential for germ cell development and the preservation of genomic integrity [63]. Another study also demonstrated that the spermatogonia-specific deletion of *Prmt5* leads to germ cell loss and male infertility [64]. *LINC00944* is another lncRNA-harboring exclusive variant that affects its structure. While this lncRNA has been associated with various cancer subtypes, most studies have focused on its role in renal cell carcinoma [65,66], where it functions as an oncogene [67]. Variants affecting structure were also found on *COX10-AS1* and *MIR663AHG*.

### 3.3. Impact of Variants on miRNA–lncRNA Interactions and Processes and the Pathways Affected

Studying miRNA–lncRNA interactions is crucial due to their significant role in gene regulation and their implications in disease development [35]. In this study, the variant rs62560775, found on *CDKN2B-AS1 (ANRIL)*, is linked to lung cancer [68]. Numerous lncRNAs with variants identified in this study, including *LINC01359* [69], *LINC01305* [70], *SLC16A1-AS1* [71,72], and *FIRRE* [73,74], are associated with cancer. *LINC01359* is also deregulated in asthenozoospermia [75] and oligozoospermia [76], whereas the host gene of *SLC16A1-AS1*, *SLC16A1*, is connected to spermatogenic defects [77]. Additionally, *FIRRE*, located on the X chromosome, plays a role in the chromosomal organization and nuclear position of the inactive X chromosome (Xi) in female cells [78].

Furthermore, regarding miRNA–lncRNA interactions, we examined the gene targets of affected miRNAs using GO and KEGG Enrichment analyses. Our findings revealed a significant presence of gene targets within the Bcl-2 protein family complex. The BCL2 protein family plays a pivotal role in apoptosis regulation and maintaining cellular balance, with members like BCL2 supporting cell survival and others like BAX promoting cell death. These proteins also regulate mitochondrial membrane permeability [79]. In the context of male infertility, our study validates prior research, highlighting the importance of BCL2 family proteins in sperm development and function [80]. Apoptosis is a dynamic process in spermatogenesis affecting germ cell divisions, differentiation, and sperm formation [81]. Imbalances in BCL2 family protein levels can disrupt spermatogenesis and fertility. In teratozoospermia, the dysregulation of the apoptotic machinery due to altered miRNA–lncRNA interactions may result in spermatozoa with abnormal morphology “es-caping” apoptosis, contributing to the condition.

KEGG pathway analysis has revealed a significant association between gene targets affected by miRNAs and cancer, reinforcing the existing link between male infertility and cancer. Previous studies have suggested a higher cancer risk in infertile men, suggesting shared molecular pathways [82,83,84,85,86,87]. Our study, supported by GO Biological Process Enrichment, identified the gene targets affected by miRNAs, including genes associated with apoptosis, programmed cell death, cell proliferation, and differentiation—all critical in cancer development [86]. Similarly, in male infertility, dysregulated apoptosis and cell processes can lead to teratozoospermia. This condition can also result from disruptions in cell proliferation and differentiation during spermatogenesis: a complex process involving spermatogonial cell growth, meiotic divisions, and spermatid maturation [88]. While common genetic pathways might link cancer and male infertility, their precise molecular mechanisms require further investigation.

Finally, GO Biological Process Enrichment and KEGG analyses identified deregulated signaling pathways in teratozoospermia, including TNF, FoxO, MAPK, and PI3K-Akt pathways. More specifically, FoxO transcription factors regulate the cell cycle, apoptosis, and oxidative stress response, impacting germ cell balance and sperm quality [89,90]. The MAPK pathway is also essential for spermatogenesis, influencing proliferation, differentiation, apoptosis, and sperm quality [91,92,93]. PI3K-Akt regulates the hypothalamus–pituitary–gonad (HPG) axis, spermatogonia, somatic cells, and sperm autophagy, affecting germ cell survival and spermatogenesis [94], while TNF, as an inflammation-related cytokine, is linked to testicular dysfunction, impaired sperm function, and increased apoptosis, all impacting sperm production and quality [95,96]. Additionally, our study revealed that many affected miRNAs target kinases, which are essential for regulating these processes and various sperm development stages in the testis [97,98].

### 3.4. Common Variants and lncRNAs with Multiple Pioritized Variants

In the present study, we also identified variants that affected both the function or structure of lncRNAs and their interactions with miRNAs, as presented in Table 3. These findings suggest the dual impact of the above variants on lncRNA functionality and interactions, potentially contributing to teratozoospermia. Therefore, these variants hold potential for future investigation as biomarkers, and conducting functional experiments could help validate their impact on the functionality and structure of lncRNAs, unveiling the molecular mechanisms underlying teratozoospermia.

Furthermore, we identified lncRNAs with multiple prioritized SNPs, as depicted in Table 4. Among the above lncRNAs, COX10 antisense RNA 1 (*COX10-AS1*) stands out as it harbors seven variants that are found only in teratozoospermic men and is the only one that has been linked in the past with male infertility as it dysregulates not only in teratozoospermia but also other types of male infertility, such as asthenozoospermia [75] and oligozoospermia [76]. While its role in human diseases was previously poorly understood, several studies have associated *COX10-AS1* with various types of human cancers, suggesting its involvement as an oncogene [99,100,101]. These studies have shed light on the complex interactions between *COX10-AS1* and miRNAs, promoting cell proliferation and inhibiting apoptosis in cancer cells [102,103]. The emerging evidence of *COX10-AS1*’s significance in both male infertility and cancer highlights its potential as a crucial player in these Biological Processes, warranting further investigation.

*CDKN2B-AS1 (ANRIL)* is also a captivating lncRNA with implications in various cancer types, where it serves as a critical regulator of cell proliferation [61,62,63]. Additionally, it has been linked to several non-malignant diseases, such as idiopathic pulmonary fibrosis, endometriosis, inflammatory bowel disease, intracranial aneurysm, diabetes mellitus, coronary artery diseases, and atherosclerosis [64]. However, intriguingly, no studies to date have associated this lncRNA with male infertility or proposed its role in spermatogenesis. Despite its extensive involvement in different diseases, the absence of investigations regarding its connection to male fertility warrants further research to explore its potential relevance in this context.

Among the identified lncRNAs mentioned above, several have also been associated with cancer. *LINC01116*, for instance, has a well-established role in cancer development, with numerous studies supporting its involvement in promoting cell proliferation, invasion, migration, and apoptosis [104]. Similarly, *HOXC-AS3* is extensively implicated in tumorigenesis through the promotion of proliferation [105,106]. *A1BG-AS1* has been linked to cancer in some studies [107,108], while *ARHGEF26-AS1* is a ferroptosis-related lncRNA with a role in tumorigenesis [109,110]. Additionally, *MIR663AHG* has been found to be downregulated in psoriatic tissues [111] and acts as a tumor suppressor, inhibiting the development of colon cancer by cis-binding to miR663a/pre-miR663a [112].

On the other hand, the available studies are limited for some lncRNAs. *RAP2C-AS1*, which has been implicated in esophageal cancer [113], *AQP4-AS1*, while also being associated with retinal neurovascular dysfunction [114], possibly cancer [102], and *DCTN1-AS1*, potentially contributing to Alzheimer’s disease development [103], has undergone scarce investigations. Furthermore, no studies explore the role and function of *STAU2-AS1* and *PRICKLE2-AS3*. Importantly, for all the mentioned lncRNAs, no studies have implicated their role in male infertility or spermatogenesis. While these lncRNAs have been linked to cancer and other diseases, their involvement in male reproductive health remains unexplored, presenting an avenue for further research to understand their potential significance in male fertility and spermatogenesis.

## 4. Materials and Methods

### 4.1. Whole Genome Sequencing (WGS)—Identification of Exclusive Variants on Teratozoospermic Men

Blood and semen samples were collected from Greek volunteers participating in the Spermogene (Fertilaid) research program (Grant number Τ1ΕΔK-02787) in collaboration with the “Embryolab IVF Unit” in Thessaloniki, Greece. This study obtained ethical approval from the Ethics Committee of the University of Thessaly, and all participants willingly provided written informed consent for their participation in the program.

All recruited volunteers underwent an andrological examination, and their semen samples were subjected to comprehensive analysis. Sperm samples were collected via masturbation, following a minimum abstinence period of two to three days. The semen analysis followed the guidelines outlined in the fifth edition (2010) of the World Health Organization (WHO). This analysis included evaluating parameters such as semen volume, sperm count, motility, morphology, etc. Cell vision counting slides (Tek-Event) were utilized for cell counting, and Nikon Eclipse TS100, E200, and Ts2 microscopes (Minato, Japan) were employed for observation during semen analysis. WHO guidelines were employed for the processing and classification of human sperm. To elaborate, the samples underwent classification using the seminogram results and reference values stipulated in the WHO guidelines. The classification categorized the samples into two groups: those with morphology falling below the 5th percentile of fertile subjects and those with morphology exceeding the 5th percentile of fertile subjects. It is important to note that this classification is in line with the latest version of the WHO guidelines, specifically the 2021 edition (accessible at https://www.who.int/publications/i/item/9789240030787, accessed on 25 August 2023). This approach replaces the use of the terms “teratozoospermic” and “normozoospermic”, respectively.

Then, genomic DNA was extracted from the blood samples of five individuals with teratozoospermia and ten individuals with normozoospermia using the PureLink Genomic DNA Mini Kit (Invitrogen, Waltham, MA, USA—Catalog number: K182002), following the manufacturer’s instructions. The quality of the DNA was assessed using agarose gel electrophoresis, while the quantity was determined using the Qubit 2.0 fluorometer and the Qubit dsDNA BR Assay Kit (Invitrogen, Waltham, MA, USA—Catalog number: Q32850). Subsequently, the DNA samples were divided into three sequencing pools. Two of the pools contained DNA from the normozoospermic individuals, with each pool consisting of DNA from five individuals. The third pool was created by pooling the DNA from teratozoospermic individuals. The DNA within each pool was mixed in equimolar ratios, resulting in a final concentration of 100 ng/µL and a total quantity of 2 mg.

After the completion of sample preparation, the DNA samples were shipped to Novogene (Cambridge, UK) for sequencing. Paired-end libraries with 100 bp reads were constructed, and sequencing was performed using an Illumina HiSeq 3000 platform, aiming for an average sequencing coverage of 30×. The quality of the generated FASTQ files was initially assessed using FASTQC [115]. Subsequently, Trimmomatic [116] was used to remove low-quality reads (with a minimum PHRED score of 30) and adapter sequences. Following quality control, the reads were aligned to the GRCh37/hg19 human reference genome obtained from the Ensembl database [117] using the Burrows–Wheeler aligner (BWA) (version 0.7.17) [118]. Duplicate reads resulting from the polymerase chain reaction (PCR) were identified and removed using Picard tools before further analysis. The alignment results were then converted from the SAM to BAM format using SAMtools [119]. Next, the individual BAM files from the two normozoospermic pools were merged into a single file representing normozoospermic individuals while also using SAMtools [119]. A variant calling was performed using freeBayes (version 1.3.6) [120], and the resulting variants were stored in the variant call format (VCF). It should be noted that for BWA and freeBayes, default parameters were used as proposed by the developers. To identify unique variants specific to either normozoospermic or teratozoospermic individuals, the VCF files from both groups were compared using BCFtools [119]. These unique variants, which were not shared between the two groups (teratozoospermic and normozoospermic), were subjected to further analysis, focusing on individuals diagnosed with teratozoospermia. These exclusive variants found in teratozoospermic patients have the potential to contribute to the pathogenic phenotype and provide valuable insights into the molecular mechanisms underlying male infertility, particularly in the context of teratozoospermia. After the detection of unique variants for teratozoospermic individuals, annotation was performed using the VEP tool [121] provided by the Ensembl database. Among the types of annotations performed, these included the genes and the transcripts affected, the type of variants (coding/non-coding), the consequence of the variants (intronic, intergenic, frameshift, missense, synonymous, etc.), and associated minor allele frequencies from the 1000 Genomes Project [122], etc.

### 4.2. RNA Expression Profiles—Identification of Differentially Expressed lncRNAs between Normozoospermic and Teratozoospermic Men

Data were retrieved from the publication of Zhou and Wang (2020) [27] to identify differentially expressed (DE) lncRNAs between teratozoospermic and normozoospermic individuals. In summary, as described in the publication of Platts et al. (2007) [123], semen samples were collected from thirteen normospermic and eight teratozoospermic men to investigate the RNA expression profiles of human spermatozoa. RNA extraction was performed on purified sperm cells obtained from ejaculate samples, and the extracted RNA was subjected to hybridization using Affymetrix U133 (v2) Microarrays. The data from this study are available in the GEO database under the accession number GSE6872 [123]. Then, based on the analysis of Zhou and Wang (2020) [27], DE lncRNAs were identified using cut-off criteria with a *p*-value < 0.05 and [logFC] > 2 [27].

### 4.3. Identification of Exclusive Variants on DE lncRNAs and Variant Prioritization—Investigation of Their Role and Consequence

Following the identification of variants exclusively found in teratozoospermic men through whole-genome sequencing analysis and the identification of differentially expressed (DE) lncRNAs between teratozoospermic and normozoospermic men, these datasets were integrated. Only the exclusive variants found in teratozoospermic men that mapped onto DE lncRNAs were selected for further analysis, as variants occurring in lncRNAs had the potential to influence the expression level, structure, and function of these lncRNAs by interfering with the expression of their corresponding target mRNAs [124]. In this way, they can contribute to the development of complex disorders, such as male infertility [21]. Additionally, these variants hold the potential to serve as valuable biomarkers [124].

Then, to further prioritize the exclusive variants on DE lncRNAs, a series of filters were applied. Specifically, variants with potential functional roles were selected based on information from the 3DSNP [29] and RegulomeDB databases [28]. RegulomeDB integrates data from ENCODE, GEO, and other sources to identify the regulatory roles of non-coding SNPs. Its main function is to assign scores to SNPs, allowing for the differentiation of functional SNPs from a large pool of variants. Each SNP is assigned a rank from 1 to 7, with lower values indicating a higher likelihood of having a regulatory function [28]. Similarly, the 3DSNP database provides information on 3D-interacting genes, enhancer states, promoter states, transcription factor binding sites, altered sequence motifs, and conservation. It calculates a functional score for each SNP, with higher scores indicating a greater likelihood of SNP functionality [29]. Thus, to assess the impact of variants on lncRNA functionality, only variants meeting the criteria of a 3DSNP score > 10 and a RegulomeDB Rank between 1a and 2c were used for further analysis. It is important to note that when evaluating the functionality of variants and prioritizing them, we opted for specific thresholds. These thresholds included a 3DSNP score greater than 10 and a RegulomeDB Rank less than 3. These thresholds were chosen based on previous and similar publications, where they have been associated with a relatively high level of evidence for the potential regulatory functions of SNPs [125,126,127,128]. Additionally, the lncRNA structure changes induced by variants were also investigated. The lncRNASNP v3 [33] database utilizes data from RNAsnp [129] to evaluate the effects of variants on lncRNA’s secondary structure. Thus, variants were filtered, retaining only those with a *p*-value < 0.2, indicating their influence on lncRNA structure for subsequent analysis. In our analysis, a *p*-value less than 0.2 meant that a variant had a notable effect on lncRNA structure. This threshold aligns with the recommendations from the creators of lncRNASNP v3 [33] and is based on calculations from RNAsnp [129]. In brief, the program computes base-pairing probabilities for both the original (wild-type or WT) and altered (alternate or ALT) sequences containing the SNP. It then assesses the structural distinction between these sequences using metrics like Euclidean distance (d) and Pearson’s correlation coefficient (r) for RNA segments spanning at least 50 nucleotides. The most significant structural change, either in terms of the maximum base pairing distance (d_max_) or minimum correlation coefficient (r_min_), is identified. Empirical *p*-values are calculated to gauge the likelihood of this change occurring randomly. If the p-value for d_max_ falls below 0.2, it indicates that the SNP induces a substantial and non-random alteration in RNA structure. Furthermore, considering that lncRNAs interact with miRNAs to regulate gene expression, the lncRNASNP v3 database [33] was employed to identify exclusive variants on teratozoospermic men that result in the gain or loss of miRNA target sites on the aforementioned DE lncRNAs. This specific database was selected as it represents the intersection of results from MiRanda, TargetScan, and Pita, providing the final miRNA targets of lncRNAs. Subsequently, the gene targets of the affected miRNAs were identified using experimental interactions obtained from miRTargetLink 2.0 [36]. To gain further insights into the role of these gene targets and identify deregulated pathways in teratozoospermia resulting from exclusive variants on DE lncRNAs that affect their interaction with miRNAs, Gene Ontology (GO) Enrichment analysis [37,38] and KEGG pathway analysis [39] were conducted using ShinyGO 0.77 [130]. For both GO and KEGG analyses, it is essential to highlight that statistical significance was reported after correcting for the false discovery rate (FDR) in order to account for multiple comparisons. Specifically, we applied an FDR-adjusted *p*-value threshold of <0.05. It is also important to note that only the overlapping gene targets of affected miRNAs were utilized for both the Gene Ontology (GO) Enrichment analysis [37,38] and the KEGG pathways analysis [39].

**Figure 2:**
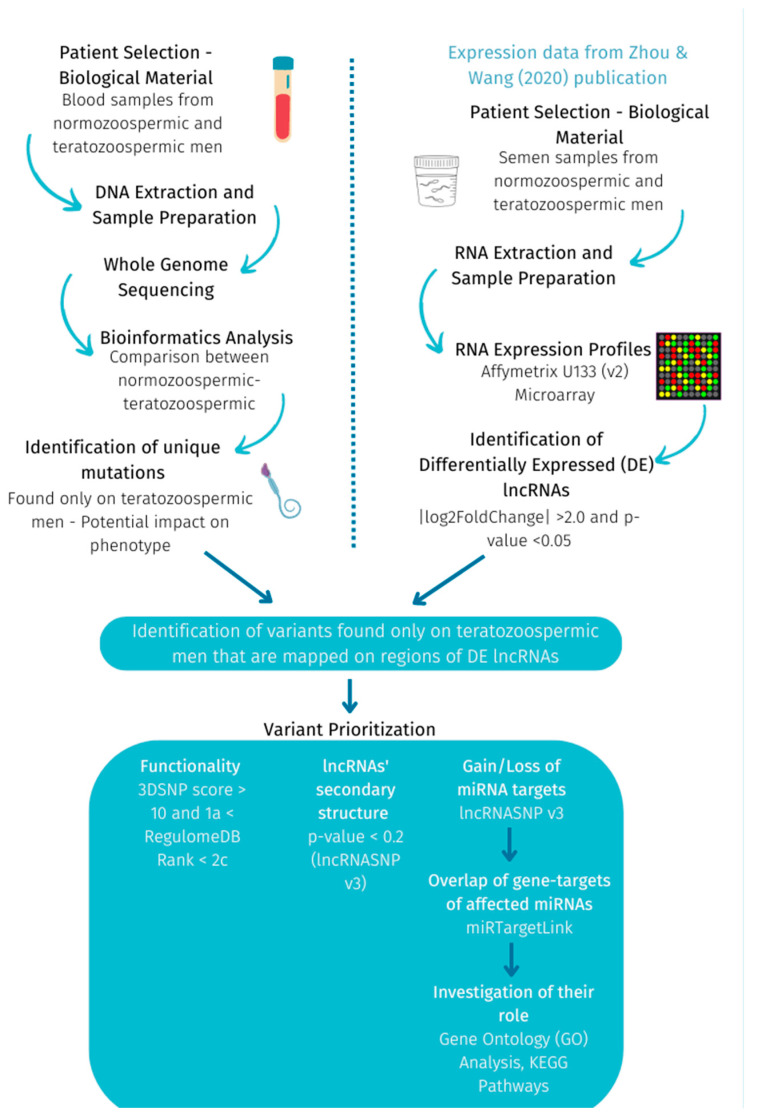
Methodology followed to investigate exclusive variants on DE lncRNAs between teratozoospermic and normozoospermic men.

In summary, the methodology used in this study is presented in Figure 2.

## 5. Conclusions

In conclusion, this study represents the first comprehensive investigation into the impact of specific variants on lncRNAs’ function and structure, utilizing whole-genome sequencing and RNA expression profiles of patients with teratozoospermia. This research is highly significant as it lays the foundation for future studies by identifying the candidate lncRNAs and variants associated with teratozoospermia for the first time. The prioritized variants found exclusively in teratozoospermic men hold the potential to serve as valuable biomarkers pending further experimentation. Moreover, the identification of a link between male infertility and cancer opens promising avenues for future research. However, the small sample size of recruited patients is a limitation of this study, and therefore, the validation of these findings in a larger sample is recommended. Additionally, functional experiments are essential to confirm the impact of these variants on the structure and function of lncRNAs, as the present study relied on bioinformatics tools. Such validations could strengthen the credibility and applicability of the study’s outcomes. However, it should be noted that we made concerted efforts to address the above-mentioned limitations by leveraging multiple databases and implementing a stringent set of criteria and filters during variant analysis.

Therefore, this study makes a significant contribution to our understanding of teratozoospermia by shedding light on important pathways that undergo deregulation due to variants on lncRNAs. The identification of specific lncRNAs and variants serves as a valuable foundation for enhancing teratozoospermia diagnosis, as a diverse range of variants and previously unexplored lncRNAs have been uncovered, holding promise as candidates for future research.

It is worth noting that this study represents a preliminary exploration into SNPs found within differentially expressed lncRNAs and potentially associated with teratozoospermia. Consequently, our findings should be interpreted with caution and validated in larger cohorts. Nevertheless, it is important to recognize that smaller-scale studies, such as the one presented here, can still yield valuable insights and lay the foundation for future research. By identifying specific SNPs and drawing attention to lncRNAs that may contribute to the pathogenesis of male infertility, especially in cases where limited information is available regarding variants affecting lncRNA’s function and structure, studies of this nature can offer valuable insights into the genetics of complex diseases and traits, such as teratozoospermia.

## Figures and Tables

**Table 1:** Prioritized variants found exclusively in teratozoospermic men, which are mapped to differentially expressed lncRNAs, and have a strong likelihood of affecting lncRNAs’ functionality, according to RegulomeDB [28] and 3DSNP [29] databases.

Variant	lncRNA	3DSNP Score	RegulomeDB Rank
rs11170045	*LINC00592*	42.35	1b
rs17126450	*LINC00592*	34.82	1b
rs7972661	*LINC00592*	28.40	1f
rs936329	*LINC00592*	26.09	1f
rs1870213	*LINC00592*	27.76	1f
rs143637901	*LINC00592*	27.63	1f
rs7301136	*LINC00592*	23.79	1f
rs7315889	*LINC00592*	44.57	1b
rs10783499	*LINC00592*	45.49	1f
rs7316130	*LINC00592*	45.37	1f
rs10876245	*LINC00592*	46.98	1f
rs10876246	*LINC00592*	14.97	1f
rs10876247	*LINC00592*	16.98	1f
rs10876248	*LINC00592*	12.32	1f
rs10783500	*LINC00592*	12.40	1f
rs7303604	*LINC00592*	10.60	1f
rs10783503	*LINC00592*	25.87	1f
rs10783504	*LINC00592*	125.41	1f
rs11170048	*LINC00592*	125.70	1f
rs11170049	*LINC00592*	125.39	1f
rs11170050	*LINC00592*	123.71	1f
rs56035420	*HOXC-AS3*	48.25	1b
rs1956568	*FRMD6-AS1*	25.01	1b
rs1956567	*FRMD6-AS1*	22.74	1f
rs566495825	*FRMD6-AS1*	16.98	2b
rs71266965	*AQP4-AS1*	23.77	2b
rs953369619	*AQP4-AS1*	38.38	2b
rs1265960	*A1BG-AS1*	23.04	1f
rs56822355	*LINC01350*	136.62	1b
rs41453048	*LINC01350*	18.39	1b
rs12029108	*LINC01350*	16.82	1f
rs72727706	*LINC01350*	12.63	1f
rs80202485	*LINC00466*	126.85	1f
rs72781367	*LINC00276*	11.09	1b
rs2969359	*LINC01116*	84.20	1b
rs2969358	*LINC01116*	84.25	1f
rs2969357	*LINC01116*	74.60	1f
rs6723379	*DCTN1-AS1*	44.15	1f
rs1727884	*ARHGEF26-AS1*	18.68	1f
rs1713828	*ARHGEF26-AS1*	55.43	1f
rs1713827	*ARHGEF26-AS1*	125.75	1f
rs71281391	*PRICKLE2-AS3*	23.94	2b
rs901028815	*LINC00877*	11.67	2b
rs59058260	*LINC00877*	15.06	2a
rs10016533	*SEC24B-AS1*	111.37	1f
rs553142360	*LINC01091*	56.16	2b
rs17491036	*LINC01091*	24.33	2b
rs1560738	*LINC01091*	21.52	1b
rs58833496	*LINC01091*	10.36	1f
rs78146337	*NNT-AS1*	12.66	1b
rs3734931	*C7orf69*	50.17	2b
rs4595031	*C7orf69*	10.44	1f
rs7823346	*ZNF252P-AS1*	205.05	2b
rs2978419	*ZNF252P-AS1*	106.11	1f
rs7832026	*ZNF252P-AS1*	54.79	1f
rs10107110	*LZTS1-AS1*	20.92	1b
rs12681283	*STAU2-AS1*	52.30	2b
rs199889712	*OTUD6B-AS1*	75.01	1b
rs3217986	*CDKN2B-AS1*	75.44	2b
rs1368574245	*CDKN2B-AS1*	92.75	2b
rs73652847	*CDKN2B-AS1*	12.19	2b
rs17694555	*CDKN2B-AS1*	14.83	1f
rs564311323	*CDKN2B-AS1*	17.41	2b
rs112111321	*RAP2C-AS1*	216.01	1a

**Table 2:** Prioritized variants found exclusively in teratozoospermic men, which are mapped to differentially expressed lncRNAs, and have an impact on the structure of lncRNAs, as determined by lncRNASNP v3 database [33].

Variant	lncRNA	Transcript	*p*-Value
rs139506376	*LINC00944*	NONHSAT031816.2	0.1688
rs200712585	*PRMT5-AS1*	NONHSAT035833.2	0.0739
	*PRMT5-AS1*	NONHSAT168171.1	0.0814
	*PRMT5-AS1*	NONHSAT168172.1	0.1409
	*PRMT5-AS1*	NONHSAT035834.2	0.0818
rs4792409	*PRMT5-AS1*	NONHSAT145734.2	0.1078
rs150364102	*COX10-AS1*	NONHSAT145753.2	0.0946
	*COX10-AS1*	NONHSAT145752.2	0.0946
	*COX10-AS1*	NONHSAT145754.2	0.0946
	*COX10-AS1*	NONHSAT145751.2	0.0921
	*COX10-AS1*	NONHSAT175957.1	0.0946
	*COX10-AS1*	NONHSAT175958.1	0.0946
rs75133618	*COX10-AS1*	NONHSAT175957.1	0.1591
	*COX10-AS1*	NONHSAT175958.1	0.1591
rs142992890	*MIR663AHG*	NONHSAT189547.1	0.1669
rs1285843394	*MIR663AHG*	NONHSAT189547.1	0.1941

**Table 3:** Exclusive variants mapped on DE lncRNAs that affect both the structure or function of lncRNAs and miRNA–lncRNA interactions.

Variants	lncRNAs	Impact on lncRNAs
rs150364102	*COX10-AS1*	Structure and miRNA–lncRNA interactions
rs56035420	*HOXC-AS3*	Function and miRNA–lncRNA interactions
rs2969359	*LINC01116*	Function and miRNA–lncRNA interactions
rs2969358	*LINC01116*	Function and miRNA–lncRNA interactions
rs2969357	*LINC01116*	Function and miRNA–lncRNA interactions
rs1713827	*ARHGEF26-AS1*	Function and miRNA–lncRNA interactions

**Table 4:** DE lncRNAs with exclusive variants that affect both their function or structure and lncRNA–miRNA interactions.

lncRNAs	Number of Variants	Impact of Variants
*COX10-AS1*	7	Structure and miRNAs–lncRNAs
*MIR663AHG*	6	Structure and miRNAs–lncRNAs
*HOXC-AS3*	2	Function and miRNAs–lncRNAs
*AQP4-AS1*	5	Function and miRNAs–lncRNAs
*A1BG-AS1*	6	Function and miRNAs–lncRNAs
*LINC01116*	7	Function and miRNAs–lncRNAs
*DCTN1-AS1*	2	Function and miRNAs–lncRNAs
*ARHGEF26-AS1*	6	Function and miRNAs–lncRNAs
*PRICKLE2-AS3*	2	Function and miRNAs–lncRNAs
*STAU2-AS1*	2	Function and miRNAs–lncRNAs
*RAP2C-AS1*	2	Function and miRNAs–lncRNAs
*CDKN2B-AS1*	7	Function and miRNAs–lncRNAs

## Data Availability

Whole-genome sequencing data presented in this study are available through SRA (BioProject ID PRJNA875412, http://www.ncbi.nlm.nih.gov/bioproject/875412) (accessed on 25 August 2023)) and RNA expression data are also available through GEO (GSE6872, https://www.ncbi.nlm.nih.gov/geo/query/acc.cgi?acc=GSE6872) (accessed on 25 August 2023)).

## Supplementary Materials

The following supporting information can be downloaded at: https://www.mdpi.com/article/10.3390/ijms241915002/s1.
